# Genome Sequencing Shows that European Isolates of *Francisella tularensis* Subspecies *tularensis* Are Almost Identical to US Laboratory Strain Schu S4

**DOI:** 10.1371/journal.pone.0000352

**Published:** 2007-04-04

**Authors:** Roy R. Chaudhuri, Chuan-Peng Ren, Leah Desmond, Gemma A. Vincent, Nigel J. Silman, John K. Brehm, Michael J. Elmore, Michael J. Hudson, Mats Forsman, Karen E. Isherwood, Darina Guryčová, Nigel P. Minton, Richard W. Titball, Mark J. Pallen, Richard Vipond

**Affiliations:** 1 Division of Immunity and Infection, University of Birmingham, Edgbaston, United Kindgom; 2 Health Protection Agency, Centre for Emergency Preparedness and Response, Porton Down, Salisbury, United Kindgom; 3 Department of NBC Analysis, Division of NBC Defence, FOI Swedish Defence Research Agency, Umeå, Sweden; 4 Defence Science and Technology Laboratory, Porton Down, Salisbury, United Kindgom; 5 Department of Epidemiology, Medical Facility, Comenius University, Bratislava, Slovak Republic; 6 Centre for Biomolecular Sciences, Institute of Infection, Immunity and Inflammation, University of Nottingham, Nottingham, United Kingdom; Massachusetts General Hospital and Harvard Medical School, United States of America

## Abstract

**Background:**

*Francisella tularensis* causes tularaemia, a life-threatening zoonosis, and has potential as a biowarfare agent. *F. tularensis* subsp. *tularensis*, which causes the most severe form of tularaemia, is usually confined to North America. However, a handful of isolates from this subspecies was obtained in the 1980s from ticks and mites from Slovakia and Austria. Our aim was to uncover the origins of these enigmatic European isolates.

**Methodology/Principal Findings:**

We determined the complete genome sequence of FSC198, a European isolate of *F. tularensis* subsp. *tularensis*, by whole-genome shotgun sequencing and compared it to that of the North American laboratory strain Schu S4. Apparent differences between the two genomes were resolved by re-sequencing discrepant loci in both strains. We found that the genome of FSC198 is almost identical to that of Schu S4, with only eight SNPs and three VNTR differences between the two sequences. Sequencing of these loci in two other European isolates of *F. tularensis* subsp. *tularensis* confirmed that all three European isolates are also closely related to, but distinct from Schu S4.

**Conclusions/Significance:**

The data presented here suggest that the Schu S4 laboratory strain is the most likely source of the European isolates of *F. tularensis* subsp. *tularensis* and indicate that anthropogenic activities, such as movement of strains or animal vectors, account for the presence of these isolates in Europe. Given the highly pathogenic nature of this subspecies, the possibility that it has become established wild in the heartland of Europe carries significant public health implications.

## Introduction

*Francisella tularensis* causes tularaemia, a potentially fatal zoonosis. Tularaemia is confined to the Northern Hemisphere, where it is maintained in the environment by rabbits, voles and other small mammals [1]. Human infection most commonly follows a bite from an arthropod that has acquired the bacterium from an infected animal. This route leads to glandular or ulceroglandular tularaemia, which is rarely fatal (∼3% mortality). Infection can also be acquired through ingestion of contaminated food or water. However, the most serious manifestation of tularaemia, with a mortality rate of up to 30%, is the respiratory form of the disease, which is acquired by inhalation of aerosolized bacteria [2], [3]. Under these circumstances as few as ten bacterial cells are sufficient to establish disease. Infectious aerosols have been generated by farming activities [4] or even by cutting grass [5], [6].

The molecular basis of *Francisella* infection remains poorly understood, largely due to a paucity of genetic tools. Recently, however, complete genome sequences from several strains have become available. The first complete genome sequence was from the strain Schu S4 [7]. This strain was originally isolated from an ulcer in a clinical case of tularaemia in Ohio in 1941 and provides an example of the highly virulent subspecies *Francisella tularensis* subsp. *tularensis*. Since its original isolation, it has been adopted widely for use in laboratory studies [8], [9].

Several subspecies of *F. tularensis* have been identified. *F. tularensis* subsp. *holarctica* (formerly Type B) is found in Europe and Asia, and to a lesser extent in North America. Several other subspecies, *mediasiatica*, *novicida* and a Japanese variant of *holarctica*, show restricted geographical ranges and play little or no role in human disease [10]. The remaining subspecies, *F. tularensis* subsp. *tularensis* (formerly Type A), is the most virulent and is usually confined to North America.

The second *F. tularensis* genome sequence (GenBank accession number AM233362) originated from the “live vaccine strain”, LVS. This strain was obtained after serial laboratory passage of a virulent *F. tularensis* subsp. *holarctica* isolate [11]. The LVS strain is known to provide protective immunity against tularaemia [12], [13]. However, as the mechanisms underlying attenuation and protection remain unclear, it is no longer licensed for use as a vaccine in the UK or USA and the search continues for a licensable vaccine against tularaemia [14]. Most recently, analysis of a third *F. tularensis* genome—like LVS from subspecies *holarctica*—has confirmed that there have been extensive genomic rearrangements since the two subspecies diverged [15].

Despite the usual finding that subspecies *tularensis* is confined to North America, several isolates from this subspecies were obtained from Europe in the 1980s. The first such isolates were recovered in 1986, during a survey of small mammals, fleas, ticks and mites in western Slovakia [16]. These isolates were identified as subspecies *tularensis* due to their ability to ferment glycerol and citrulline, high sensitivity to erythromycin and high virulence; these properties are typical of subspecies *tularensis* but not subspecies *holarctica*. Over the following two years, isolates of *F. tularensis* subspecies *tularensis* were recovered repeatedly from fleas and mites captured in the region of the Danube river basin, close to Bratislava.

Two of the isolates of *F. tularensis* subspecies *tularensis* that were recovered from Slovakia were deposited in the Swedish Defence Research Agency *Francisella* culture collection as FSC198 and FSC199. A further isolate of the same subspecies, Sev-23, was obtained during a later survey from *Ixodes* spp. ticks in South East Austria in 1990 (D. Guryčová, unpublished). We sought to clarify the relationship between the European isolates of *F. tularensis* subspecies *tularensis* and other members of this subspecies, particularly the genome-sequenced strain Schu S4, by determining the complete genome sequence of Slovakian isolate FSC198.

## Materials and Methods

One hundred and nineteen primer pairs were designed for whole-genome PCR scanning [17], applying GenoFrag [18] to the Schu S4 genome sequence. Primers pairs were designed to amplify fragments of about 17 kb that overlapped by around 100 bases. Amplification was performed as previously described [19].

For shotgun sequencing, chromosomal DNA from strain FSC198 was prepared as previously described [7]. DNA fragments 1.6–1.8 kb in size were ligated into the pLEXX AK double-insert vector and transformed into electrocompetent *E. coli* cells as directed by the manufacturer (Cloneplex AK kit, Lucigen Inc.). Purified plasmids were sequenced with each of the four primers from the pLEXX AK double insert vector. Sequencing and clean-up reactions were automated using an MWG Robosmart, and the DNA sequence was analyzed using an ABI 3700 PRISM DNA sequence analyzer (Applied Biosystems).

Bases were called from the shotgun traces using Phred [20], [21]. Obtaining read pairs is not straightforward when using the pLEXX AK double-insert vector, as the kanamycin cassette that separates the inserts can religate in either orientation, meaning that reads from the four primers could be paired in two alternative combinations. To resolve this ambiguity we adopted a comparative approach, using the program nucmer from the MUMmer package [22] to map the reads to the genome sequence of Schu S4. One of the combinations was considered correct if the sequences from at least one of the potential primer pairs could be unambiguously placed in positions consistent with pairing (on opposite strands within 3 kb of each other), and the same was not true of the alternate combination. A large proportion of read pairs could be determined in this way, the remainder were treated as unpaired reads during the assembly process.

Assembly was performed using two comparative methods. The first used the AMOScmp pipeline [23], a comparative approach that performs the initial tiling based on a reference sequence, then uses information on read pairs to resolve ambiguities. This approach is particularly suited to projects such as this, where a close relative genome is available. The second approach used Phrap, but included the Schu S4 genome as a fake read to guide initial assembly. The fake read was removed from the assembly following this initial process. Manual inspection and refinement of the assembly was performed using Consed [24]. Finishing was performed by a series of gap-closing PCRs, with primers designed using Primer3 [25] via a BioPerl [26] interface. A final assembly was obtained using AMOScmp. Reads from repetitive regions that could not be unambiguously placed based on their sequence or pairing information were randomly distributed between the copies of the repeat. To confirm the sequence of these repetitive regions, each was separately amplified and re-sequenced. The large repeat regions within the genome (two copies of the 33.9 kb pathogenicity island, together with three copies of a ∼3 kb repeat region consisting of a gene with no known homologues, flanked by an ISFtu1 element and an ISFtu2 element) were resolved by long PCR amplification of the entire repeats (using two overlapping long PCRs in the case of the 33.9 kb islands). Fragments from within the long PCR products were amplified, sequenced and assembled using Phrap to unambiguously determine the sequence of each repeat.

Upon final assembly, whole genome comparison of FSC198 and Schu S4 was performed using the run-mummer3 component of MUMmer version 3.0 [22], [27], and visualized using ACT [28]. For further analysis an online comparative genomics database, *Ft*BASE (http://ft.bham.ac.uk), was developed based on the *x*BASE [29] template. As only a small number of differences were identified between the two sequences, all relevant regions were PCR-amplified and re-sequenced in both the FSC198 and Schu S4 genomes, to determine if the differences were real or artefacts introduced during the sequencing, base-calling or assembly process.

Given the similarity of the two sequences, a fresh annotation of the FSC198 genome was deemed unnecessary. Instead, gene predictions and annotation were transferred from the Schu S4 genome, with features that overlapped SNPs and/or indels adjusted as necessary. The completed genome sequence has been deposited in EMBL and assigned the accession number AM286280.

To examine the diversity of the European subspecies *tularensis* isolates, the SNPs and the VNTR regions identified in this and previous studies [30] were also sequenced in strains FSC199 and Sev-23 and compared with the equivalent LVS and Schu S4 sequences.

## Results

**Figure 1:**
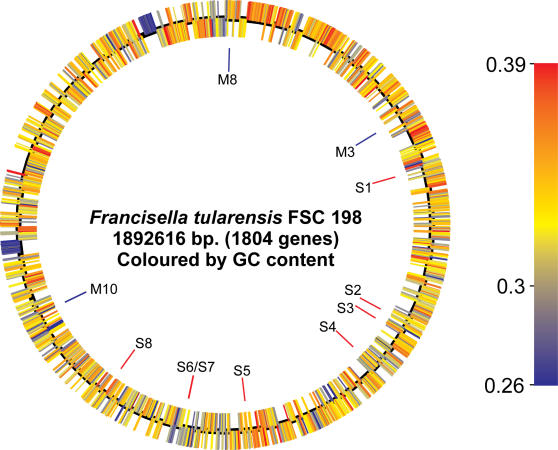
Circular representation of the complete genome sequence of FSC198. Predicted coding sequences are colored according to their GC content. The inner circle indicates the positions of SNPs (red) and VNTR differences (blue) relative to the published Schu S4 genome sequence. SNP and VNTR loci are numbered as in table 1.

All but two of 119 primer pairs patterned on the Schu S4 genome sequence yielded appropriately sized fragments when applied to DNA from FSC198 in whole-genome long PCR scanning. This confirmed that the genomes are essentially co-linear. Subsequent determination of the complete genome sequence of FSC198 showed, surprisingly, that it was almost identical to that of Schu S4 (Figure 1). Although our initial analysis of the completed FSC198 genome sequence suggested that SchuS4 and FSC198 differed at forty SNPs (Single Nucleotide Polymorphisms) and five VNTR loci, the majority of these apparent discrepancies were confirmed as errors in the published Schu S4 sequence rather than genuine differences (see Table S1). Our final analysis revealed just eight SNPs and three VNTR differences between the two genomes (Table 1).

**Table 1:** SNPs and VNTR differences between the FSC198 and Schu S4 genome sequence.

Region of difference	Schu S4 coordinate	FSC 198 coordinate	Schu S4 (USA)	FSC198 (Slovakia)	FSC199 (Slovakia)	Sev-23 (Austria)	LVS (subsp. *holarctica*)
S1	390291	390243	**C**	*T*	*T*	*T*	**C**
S2	621878	621830	**C**	*T*	**C**	**C**	**C**
S3	639511	639463	**C**	*A*	**C**	**C**	**C**
S4	701628	701580	**G**	*T*	*T*	*T*	**G**
S5	911511	911463	**C**	*T*	*T*	*T*	**C**
S6	1007564	1007516	**G**	*C*	**G**	**G**	**G**
S7	1008149	1008101	**G**	*A*	**G**	**G**	**G**
S8	1134418	1134369	**G**	*A*	**G**	**G**	**G**
M8	8266	8266	**4**	*5*	*5*	*5*	2
M3	308635	308650	**21**	*14*	25	28	13
M10	1283659	1283610	**18**	*11*	*11*	10	2

Data from equivalent loci in strains FSC199, Sev-23 and LVS are also shown. Schu S4-like ancestral character states are highlighted in bold; FSC198-like character states in italics. VNTR loci are numbered as in reference 19; the data indicate the number of copies of the repeat unit.

The presence of identical residues at all eight SNP loci in Schu S4 and in another genome-sequenced strain, LVS (which belongs to an entirely different subspecies), suggests that the Schu S4 sequences represent the ancestral state for the species (Table 1). Three of the eight SNPs that distinguish FSC198 from Schu S4 are also conserved in the other two European isolates, suggesting that all three European isolates share a common ancestor that post-dates their divergence from the genome-sequenced strain of Schu S4. In other words, the most parsimonious explanation for these data is that the differences between Schu S4 and the European strains are due to substitutions within the European lineage, subsequent to their divergence from Schu S4.

## Discussion

Since their original isolation, the European isolates of *F. tularensis* subspecies *tularensis* have remained an enigma, representing striking counter-examples to the otherwise well-founded belief that this highly virulent subspecies is confined to North America [16]. We thus decided to investigate one of these isolates by genome sequencing.

While genome sequencing was underway, an analysis of VNTR patterns by another laboratory established that there was marked genomic variation within subspecies *tularensis*, sufficient to split the sub-species into two clades, A.I and A.II, each of which in turn shows notable diversity. This study also established that the European isolates fall within the A.I sub-population, which is found predominantly in the American mid-West [30], [31]. However, surprisingly, this study showed that the European isolates are the closest relatives of the laboratory strain Schu S4 [30], far closer to it than any other isolates from the mid-West or elsewhere in North America. The complete genome sequence of strain FSC198 that we describe here extends the conclusion from the earlier study, showing that FSC198 is almost identical to the previously sequenced Schu S4 strain.

What is the explanation for this close relationship between FSC198 and SchuS4? One possibility is that it reflects anthropogenic transfer of a naturally occurring representative of the A.I sub-population from the American Mid-West to central Europe. In support of this idea, Farlow *et al.* [31] recently suggested two potential modes of spread of for the A.I clade within the continental USA: (a) the transport of dogs, and, with them *Francisella*-infected dog fleas, as an explanation for the spread of the A.I sub-population from the central USA to California and (b) the deliberate mass introduction of cotton-tailed rabbits for sporting purposes as the cause of the spread of tularaemia to New England. Both modes of spread might account for the transit of A.I strains to Europe, particularly now that wild populations of Eastern cottontails (*Sylvilagus floridanus*) have become established in several parts of Europe [32]. However, previously published VNTR results plus the data presented in Table 1 suggest that this possibility is unlikely. Were a wild relative of Schu S4 to be the progenitor of the European strains, it would be expected to differ at some positions from Schu S4. Since there are no SNPs that are specific to Schu S4, we conclude that it most likely represents the immediate precursor of the European strains.

This conclusion suggests an alternative hypothesis, that FSC198 and the other European subspecies *tularensis* strains are derived from a laboratory stock of Schu S4, a widely disseminated model strain. This notion is supported by the near identity of the FSC198 and Schu S4 genome sequences and by the evidence from VNTR typing, which identifies Schu S4 as the closest relative of the European isolates. Although one cannot completely discount the potential for laboratory error in strain propagation, the fact that the three European isolates are distinct from each another and were obtained at different times suggests that they are unlikely to have arisen from repeated laboratory contamination from a single stock of Schu S4. The possibility of repeated contamination with related isolates generated by subculturing remains, but is in our opinion unlikely. An alternate possibility is that the strains represent genuine examples of a wild population of Schu S4-like bacteria. If this population is derived from Schu S4, the most likely explanation is inadvertent contamination of the environment with laboratory-derived bacteria. Such contamination could be the consequence of disposal of laboratory waste or could even result from escape of mammals or arthropods that have been infected in the laboratory in North America, or in Europe.

All the SNPs in the FSC198 genome occur within protein-coding regions and all are non-synonymous. This hints at the possibility of positive selection driving adaptation to a new environment, whether replication in the laboratory or survival in a new environmental niche in Europe. Interestingly, two of the SNPs were identified within the same gene, *ybhO*, which encodes a cardiolipin synthetase. Knockout mutations within a homologous gene from *E. coli*, *cls*, result in increased doubling times, a lower final cell density, a loss of viability in stationary phase, and several other pleiotropic effects [33]. It is therefore conceivable that changes in the sequence of the equivalent gene in *F. tularensis* could confer a selective advantage under certain conditions.

The data presented here suggest that the Schu S4 laboratory strain is the most likely source of the reported European isolates of *F. tularensis* subspecies *tularensis* and indicate that anthropogenic activities, such as movement of strains or animal vectors, account for the presence of these isolates in Europe. Given the highly pathogenic nature of this subspecies, the possibility that it has become established wild in the heartland of Europe carries significant public health implications. We suggest that the threat posed by this hazardous organism requires further environmental sampling to assess the distribution and prevalence of this subspecies in Europe.

Further more detailed epidemiological studies on other A.I strains, such as SNP discovery and even additional genome sequencing, will be required to establish beyond all doubt whether SchuS4 is indeed the progenitor of FSC198, or whether transfer of a naturally occurring close relative of SchuS4 might account for these findings. Nonetheless, this study provides a salient example of the utility of bacterial whole-genome sequencing for the purposes of public health epidemiology and also presents the first publicly available bacterial genome sequence to be determined in the United Kingdom outside of the Wellcome Trust Sanger Institute. The establishment of an independent bacterial-genome-sequencing facility within the Health Protection Agency will prove an invaluable resource in monitoring and preventing infectious disease within the United Kingdom.

## Supporting Information

Table S1 Differences between Schu S4 and FSC198 that are attributable to sequencing errors in the published Schu S4 genome sequence.
(0.05 MB DOC)

## References

[1] Ellis J, Oyston PC, Green M, Titball RW (2002). Tularaemia.. Clin Microbiol Rev.

[2] Dienst FT (1963). Tularaemia: a perusal of three hundred thirty-nine cases.. J La State Med Soc.

[3] Riley RL, Mills CC, Nyka W, Weinstock N, Storey PB (1959). Aerial dissemination of pulmonary tuberculosis. A two-year study of contagion in a tuberculosis ward.. Am J Epidemiol.

[4] Syrjala H, Kujala P, Myllyla V, Salminen A (1985). Airborne transmission of tularaemia in farmers.. Scand J Infect Dis.

[5] Feldman KA, Stiles-Enos D, Julian K, Matyas BT, Telford SR (2003). Tularaemia on Martha's Vineyard: seroprevalence and occupational risk.. Emerg Infect Dis.

[6] Feldman KA, Enscore RE, Lathrop SL, Matyas BT, McGuill M (2001). An Outbreak of Primary Pneumonic Tularaemia on Martha's Vineyard.. N Engl J Med.

[7] Larsson P, Oyston PC, Chain P, Chu MC, Duffield M (2005). The complete genome sequence of Francisella tularensis, the causative agent of tularaemia.. Nat Genet.

[8] Hesselbrock W, Foshay L (1945). The Morphology of Bacterium tularense.. J Bacteriol.

[9] Eigelsbach HT, Braun W, Herring RD (1951). Studies on the variation of Bacterium tularense.. J Bacteriol.

[10] Whipp MJ, Davis JM, Lum G, de Boer J, Zhou Y (2003). Characterization of a *novicida*-like subspecies of *Francisella tularensis* isolated in Australia.. J Med Microbiol.

[11] Eigelsbach HT, Downs CM (1961). Prophylactic effectiveness of live and killed tularaemia vaccines. I. Production of vaccine and evaluation in the white mouse and guinea pig.. J Immunol.

[12] Tarnvik A (1989). Nature of protective immunity to Francisella tularensis.. Rev Infect Dis.

[13] Sandstrom G (1994). The tularaemia vaccine.. J Chem Technol Biotechnol.

[14] Oyston PC, Sjostedt A, Titball RW (2004). Tularaemia: bioterrorism defence renews interest in Francisella tularensis.. Nat Rev Microbiol.

[15] Petrosino JF, Xiang Q, Karpathy SE, Jiang H, Yerrapragada S (2006). Chromosome rearrangement and diversification of Francisella tularensis revealed by the type B (OSU18) genome sequence.. J Bacteriol.

[16] Gurycova D (1998). First isolation of *Francisella tularensis* subsp. tularensis in Europe.. Eur J Epidemiol.

[17] Ohnishi M, Terajima J, Kurokawa K, Nakayama K, Murata T (2002). Genomic diversity of enterohemorrhagic *Escherichia coli* O157 revealed by whole genome PCR scanning.. Proc Natl Acad Sci U S A.

[18] Ben Zakour N, Gautier M, Andonov R, Lavenier D, Cochet MF (2004). GenoFrag: software to design primers optimized for whole genome scanning by long-range PCR amplification.. Nucleic Acids Res.

[19] Ren CP, Chaudhuri RR, Fivian A, Bailey CM, Antonio M (2004). The ETT2 gene cluster, encoding a second type III secretion system from *Escherichia coli*, is present in the majority of strains but has undergone widespread mutational attrition.. J Bacteriol.

[20] Ewing B, Green P (1998). Base-calling of automated sequencer traces using phred. II. Error probabilities.. Genome Res.

[21] Ewing B, Hillier L, Wendl MC, Green P (1998). Base-calling of automated sequencer traces using phred. I. Accuracy assessment.. Genome Res.

[22] Delcher AL, Phillippy A, Carlton J, Salzberg SL (2002). Fast algorithms for large-scale genome alignment and comparison.. Nucleic Acids Res.

[23] Pop M, Phillippy A, Delcher AL, Salzberg SL (2004). Comparative genome assembly.. Brief Bioinform.

[24] Gordon D, Abajian C, Green P (1998). Consed: a graphical tool for sequence finishing.. Genome Res.

[25] Rozen S, Skaletsky H (2000). Primer3 on the WWW for general users and for biologist programmers.. Methods Mol Biol.

[26] Stajich JE, Block D, Boulez K, Brenner SE, Chervitz SA (2002). The Bioperl toolkit: Perl modules for the life sciences.. Genome Res.

[27] Kurtz S, Phillippy A, Delcher AL, Smoot M, Shumway M (2004). Versatile and open software for comparing large genomes.. Genome Biol.

[28] Carver TJ, Rutherford KM, Berriman M, Rajandream MA, Barrell BG (2005). ACT: the Artemis Comparison Tool.. Bioinformatics.

[29] Chaudhuri RR, Pallen MJ (2006). *x*BASE, a collection of online databases for bacterial comparative genomics.. Nucleic Acids Res.

[30] Johansson A, Farlow J, Larsson P, Dukerich M, Chambers E (2004). Worldwide genetic relationships among *Francisella tularensis* isolates determined by multiple-locus variable-number tandem repeat analysis.. J Bacteriol.

[31] Farlow J, Wagner DM, Dukerich M, Stanley M, Chu M (2005). Francisella tularensis in the United States.. Emerg Infect Dis.

[32] Directorate of Culture and Cultural and Natural Heritage CoE..

[33] Tropp BE (1997). Cardiolipin synthase from Escherichia coli.. Biochim Biophys Acta.

