# Supplementary material for: Genome Sequencing Shows that European Isolates of Francisella tularensis Subspecies tularensis Are Almost Identical to US Laboratory Strain Schu S4
Source: PLoS One. 2007 Apr 4;2(4):e352. doi: 10.1371/journal.pone.0000352 (PMC1832225; doi:10.1371/journal.pone.0000352)
Supplement: Table S1 — Differences between Schu S4 and FSC198 that are attributable to sequencing errors in the published Schu S4 genome sequence. (0.05 MB DOC) [file pone.0000352.s001.doc]

**Table S1:** Differences between Schu S4 and FSC198 that are attributable to sequencing errors in the published Schu S4 genome sequence.

SNPs:

| Schu S4 coordinate | FSC 198 coordinate | Schu S4 base | FSC 198 base | Effect |
| --- | --- | --- | --- | --- |
| 231569 | 231584 | C | – | FTT0214 removes frameshift |
| 384701 | 384653 | C | A | Intergenic |
| 386671 | 386623 | G | T | FTT0384c substitution Q→K |
| 632388 | 632340 | G | T | Intergenic |
| 827069 | 827021 | C | A | FTT0807 substitution S→Y |
| 1057741 | 1057692 | T | – | Intergenic |
| 1058263 | 1058214 | T | C | FTT1047c substitution T→A |
| 1058383 | 1058334 | C | T | FTT1048c substitution A→T |
| 1058471 | 1058422 | T | C | FTT1048c synonymous |
| 1258096 | 1258047 | A | G | FTT1238c synonymous |
| 1260364 | 1260315 | T | C | FTT1240c substitution H→R |
| 1260478 | 1260429 | A | C | FTT1240c substitution I→R |
| 1310979 | 1310788 | A | – | Within 16S–rRNA |
| 1311616 | 1311425 | C | G | Within 16S–rRNA |
| 1311617 | 1311426 | G | C | Within 16S–rRNA |
| 1312265 | 1312074 | C | G | Within 16S–rRNA |
| 1312266 | 1312075 | G | C | Within 16S–rRNA |
| 1313294 | 1313102 | T | – | FTT1289 introduces frameshift |
| 1332327 | 1332134 | A | – | FTT1309c (ISFtu1) Close to stop codon, frameshift results in extended ORF2 |
| 1356837 | 1356643 | C | – | FTT1328c removes frameshift |
| 1429939 | 1429745 | T | C | FTT1385c substitution O→R |
| 1530604 | 1530410 | C | G | kdsB substitution S→T |
| 1684058 | 1683854 | C | G | FTT1620 (ISFtu1) substitution Q→E |
| 1684161 | 1683957 | A | G | FTT1620 (ISFtu1) substitution K→S |
| 1684162 | 1683958 | G | T | FTT1620 (ISFtu1) substitution K→S |
| 1684163 | 1683959 | C | T | FTT1620 (ISFtu1) substitution L→F |
| 1684165 | 1683961 | G | C | FTT1620 (ISFtu1) substitution L→F |
| 1684166 | 1683962 | T | C | FTT1620 (ISFtu1) substitution Y→H |
| 1684176 | 1683972 | A | C | FTT1620 (ISFtu1) substitution Y→S |
| 1708126 | 1707923 | – | A | FTT1643 (ISFtu1) removes frameshift |
| 1766214 | 1766011 | G | A | FTT1697c (ISFtu1) substitution A→V |
| 1845274 | 1845071 | T | A | FTT1756c (ISFtu1) synonymous |

VNTRs:

| Schu S4 coordinate | FSC 198 coordinate | Repeat Unit | Schu S4 copy no. | FSC 198 copy no. |
| --- | --- | --- | --- | --- |
| 1283964 | 1283803 | aaaatgccatcatatagccaagattttag | 2 | 1 |
| 1675401 | 1675207 | gtagaaattg | 2 | 1 |
